# A Small-Molecule Tankyrase Inhibitor Reduces Glioma Stem Cell Proliferation and Sphere Formation

**DOI:** 10.3390/cancers12061630

**Published:** 2020-06-19

**Authors:** Kirsten Strømme Kierulf-Vieira, Cecilie Jonsgar Sandberg, Jo Waaler, Kaja Lund, Erlend Skaga, Birthe Mikkelsen Saberniak, Ioannis Panagopoulos, Petter Brandal, Stefan Krauss, Iver Arne Langmoen, Einar Osland Vik-Mo

**Affiliations:** 1 Vilhelm Magnus Laboratory for Neurosurgical Research, Institute for Surgical Research and Department of Neurosurgery, Oslo University Hospital, P.O. Box 4950 Nydalen, 0424 Oslo, Norway; cecilie.sandberg@rr-research.no (C.J.S.); erlend.skaga@gmail.com (E.S.); Birthe.Mikkelsen.Saberniak@rr-research.no (B.M.S.); i.a.langmoen@medisin.uio.no (I.A.L.); Einar.Vik-Mo@rr-research.no (E.O.V.-M.); 2 Norwegian Stem Cell Center, Oslo University Hospital, University of Oslo, P.O. Box 1112 Blindern, 0317 Oslo, Norway; 3 Institute of Clinical Medicine, Faculty of Medicine, University of Oslo, P.O. Box 1112 Blindern, 0317 Oslo, Norway; 4 Department of Immunology and Transfusion Medicine, Oslo University Hospital, P.O. Box 4950 Nydalen, 0424 Oslo, Norway; Jo.Waaler@rr-research.no (J.W.); kaja90@hotmail.com (K.L.); Stefan.KRAUSS@rr-research.no (S.K.); 5 Hybrid Technology Hub-Centre of Excellence, Institute of Basic Medical Sciences, University of Oslo, P.O. Box 1110 Blindern, 0317 OSLO, Norway; 6 Section for Cancer Cytogenetics, Institute for Cancer Genetics and Informatics, The Norwegian Radium Hospital, Oslo University Hospital, Montebello, P.O. Box 49534 Nydalen, 0424 Oslo, Norway; Ioannis.Panagopoulos@rr-research.no (I.P.); petter.brandal@ous-hf.no (P.B.); 7 Department of Oncology, The Norwegian Radium Hospital, Oslo University Hospital, Montebello, P.O. Box 49534 Nydalen, 0424 Oslo, Norway; 8 Centre for Cancer Biomedicine, Faculty of Medicine, University of Oslo, P.O. Box 1112 Blindern, 0317 Oslo, Norway; 9 Department of Neurosurgery, Oslo University Hospital, P.O. Box 4950 Nydalen, 0424 Oslo, Norway

**Keywords:** glioblastoma, glioma stem cells, tankyrase, temozolomide, β-catenin, WNT, Hippo

## Abstract

Evidence suggests that the growth and therapeutic resistance of glioblastoma (GBM) may be enabled by a population of glioma stem cells (GSCs) that are regulated by typical stem cell pathways, including the WNT/β-catenin signaling pathway. We wanted to explore the effect of treating GSCs with a small-molecule inhibitor of tankyrase, G007-LK, which has been shown to be a potent modulator of the WNT/β-catenin and Hippo pathways in colon cancer. Four primary GSC cultures and two primary adult neural stem cell cultures were treated with G007-LK and subsequently evaluated through the measurement of growth characteristics, as well as the expression of WNT/β-catenin and Hippo signaling pathway-related proteins and genes. Treatment with G007-LK decreased in vitro proliferation and sphere formation in all four primary GSC cultures in a dose-dependent manner. G007-LK treatment altered the expression of key downstream WNT/β-catenin and Hippo signaling pathway-related proteins and genes. Finally, cotreatment with the established GBM chemotherapeutic compound temozolomide (TMZ) led to an additive reduction in sphere formation, suggesting that WNT/β-catenin signaling may contribute to TMZ resistance. These observations suggest that tankyrase inhibition may serve as a supplement to current GBM therapy, although more work is needed to determine the exact downstream mechanisms involved.

## 1. Introduction

Glioblastoma (GBM) is the most common and malignant central nervous system (CNS) tumor in adults. The prognosis for GBM is invariably poor; despite intensive treatment comprising surgery, irradiation, and chemotherapy, the median survival in unselected material is less than one year [1]. Subpopulations of highly aggressive, therapy-resistant tumor cells, labeled glioma stem cells (GSCs), are believed to be drivers of GBM’s malignant behavior [2,3,4].

GSCs can be propagated and enriched in cell aggregates known as tumor spheres under serum-free, growth factor-enriched conditions. Cells from these spheres share similarities with adult human neural stem cells (ahNSCs) in their capacity for long-term self-renewal and in their ability to differentiate into the various cell types of the CNS [5,6]. The ability to form tumor spheres from GBM biopsies has been shown to be an independent predictor of patient outcomes [7,8]. Upon transplantation into rodents, GSCs produce invasive tumors that are histologically similar to their tumor of origin [3].

A growing body of evidence suggests that the WNT/β-catenin signaling pathway is important for the development and regulation of cancer stem cells (CSCs), including GSCs [9,10,11,12,13]. WNT/β-catenin signaling is controlled both by the level and activity of WNT pathway proteins [14] as well as by other pathways and enzymes [15]. Tankyrases (TNKS) are enzymes that regulate a variety of cellular functions, including telomere homeostasis, mitotic spindle formation, vesicle transport, energy metabolism and several signaling pathways, such as WNT/β-catenin, Hippo, PI3K/AKT and AMPK [16,17]. In WNT/β-catenin signaling, TNKS act through the poly(ADP-ribosyl)ation of AXIN1 and AXIN2 (AXIN1/2), the rate-limiting structural proteins in the β-catenin degradosome that control the turnover of the transcriptional regulator β-catenin [18]. Poly(ADP-ribosyl)ation targets AXIN1/2 for ubiquitination and proteasomal degradation. The blockade of TNKS enzymatic activity results in the accumulation of AXIN1/2-containing β-catenin degradosomes and reduced WNT/β-catenin signaling activity [18,19]. The development of TNKS inhibitors has received focus because of their potential as a possible anticancer treatment strategy, and chemical TNKS inhibition has been shown to impact a number of tumor models [20,21,22,23].

Studies of TNKS inhibitors in cancer cells, including G007-LK [20,24], have shown that inhibiting TNKS regulates Hippo signaling [25,26,27]. The Hippo signaling pathway plays fundamental roles in tissue homeostasis and organ size control, and the pathway involves kinase cascades, adaptor proteins and the downstream oncogenes TAZ and YAP, which bind to nuclear transcription factors to activate signaling [28]. Active Hippo signaling regulates the kinases LATS1 and LATS2, which phosphorylate TAZ and YAP in the cytoplasm, marking them for proteasomal degradation, resulting in tumor suppressor activity [28]. TNKS inhibitors impede YAP/TAZ oncogenic activity by stabilizing adaptor proteins from the angiomotin (AMOT) family that sequester YAP to the cytoplasm, thus preventing YAP-driven target gene activation [26,29].

We have previously demonstrated that restoration of WNT inhibition through treatment with the WNT inhibitor SFRP1 reduces GSC tumorigenicity through the modification of essential CSC pathways [10]. In the present study, we explored the effectiveness and mechanism of action of G007-LK on primary GSC cultures. We found that G007-LK treatment reduced proliferation and sphere formation in GSCs and affected the expression of key proteins and genes in the WNT/β-catenin and Hippo signaling pathways. Moreover, cotreatment with G007-LK and the GBM chemotherapy drug temozolomide (TMZ) reduced sphere formation but not proliferation compared to treatment with TMZ alone.

## 2. Results

### 2.1. Treatment with G007-LK Attenuates GSC Growth at Levels Comparable with Those for a Colon Cancer Cell Line

**Figure 1:**
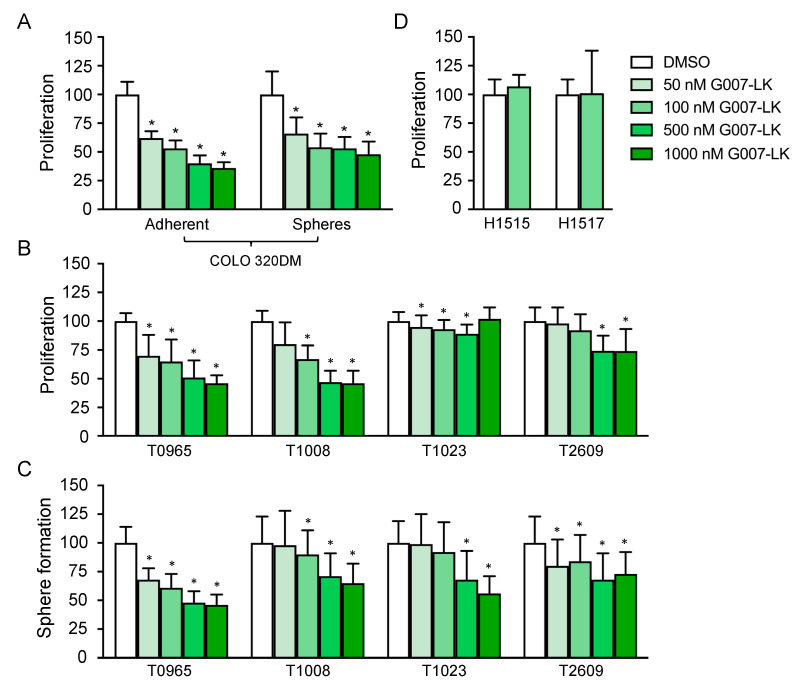
G007-LK reduces proliferation and sphere formation in primary glioma stem cell (GSC) cultures. (**A**) Proliferation of COLO 320DM cells treated with G007-LK or DMSO (0.01%) using both adherent (left panel) and sphere-forming (right panel) culture conditions; (**B**) Proliferation of GSCs treated with G007-LK or DMSO (0.01%); (**C**) Sphere formation of GSCs treated with G007-LK or DMSO (0.01%); (**D**) Proliferation of ahNSCs treated with G007-LK (100 nM) or DMSO (0.002%). Proliferation was measured by XTT assays and sphere formation as the total number of spheres formed in sphere formation assays, both after 14 days of treatment. Values are expressed as percentages relative to the DMSO control. The results are presented as the mean ± SD. * *p* < 0.05.

The effect of tankyrase inhibition through WNT/β-catenin inhibition was initially documented in colon cancer cell lines [30]. Colon cancers are known to be highly WNT-dependent as a result of mutations in adenomatous polyposis coli (APC), leading to the nuclear accumulation of β-catenin and constitutive WNT/β-catenin signaling [31]. Such mutations are relatively rare in GBM [32]. Instead, we have described the epigenetic silencing of WNT inhibitors as a pathway-enhancing mechanism in malignant brain cancer [10,33]. We therefore sought to compare the sensitivity of GSCs to G007-LK with that of a highly WNT/β-catenin-dependent cell line. Thus, we first evaluated the effect of G007-LK on the APC-mutated colon cancer cell line COLO 320DM. To ensure a functional comparison between COLO 320DM cells and primary GSC cultures, we measured G007-LK’s anti-proliferative effect in both adherent and sphere-forming culture conditions. The reduction in proliferation after 14 days was substantial and dose-dependent; there was a significant reduction at the lowest concentration of G007-LK (50 nM) and a maximum reduction of more than 50% at the highest concentration (1 µM; Figure 1a). The anti-proliferative effect of G007-LK was not significantly different between the two culture conditions.

GSCs were established from four primary GBMs—T0965, T1008, T1023 and T2609—and they were cultured as spheres. We have previously verified that these cultures express stem cell markers (SOX2 and CD133), have the ability to differentiate upon the removal of growth factors, and form tumors upon orthotopic xenografting [8,33,34,35]. To assess the anti-proliferative effect of G007-LK, the four GSC cultures were treated with G007-LK under sphere-forming culture conditions for 14 days. Similar to the anti-proliferative effect seen in COLO 320DM cells (Figure 1a), a dose-dependent reduction in proliferation was observed, reaching more than 50% at the highest concentration used (1 µM) in the two most sensitive cultures (T0965 and T1008; Figure 1b). A similar pattern was observed for sphere formation, and the reduction was above 50% at the highest concentration used (1 µM) in the most sensitive culture, T0965 (Figure 1c).

To evaluate the possible adverse effects on normal cell populations, G007-LK was tested on two primary ahNSC cultures. The proliferation of both cultures was unaffected by a 14-day treatment with 100 nM G007-LK (Figure 1d), a concentration at which the G007-LK-sensitive GSC cultures showed a clear anti-proliferative response.

### 2.2. G007-LK Stabilizes Cytoplasmic AXIN1 and Reduces the Expression of WNT/β-Catenin Target Genes

**Figure 2:**
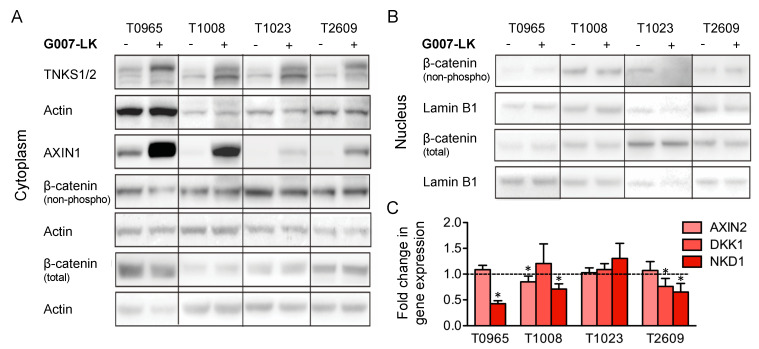
G007-LK stabilizes cytoplasmic AXIN1 and reduces the expression of WNT/β-catenin target genes. The effect of G007-LK treatment on the (**A**) cytoplasmic and (**B**) nuclear levels of WNT/β-catenin signaling proteins, as assessed by Western blotting; (**C**) Fold change in gene expression of WNT/β-catenin target genes, as assessed by qPCR upon treatment with G007-LK. For both analyses, GSC cultures were treated for 72 h with G007-LK (500 nM) or DMSO (0.01%). Values are relative to those of the DMSO control and are expressed as the fold changes from the DMSO control. DKK1 was not detectable in T0965 and is therefore not shown. The results are presented as the mean ± SD. * *p* < 0.05.

G007-LK has been shown to inhibit WNT/β-catenin signaling in a cell type- and context-dependent manner that varies between cell cultures [17,20]. Therefore, we examined the effect of G007-LK on central biotargets in the WNT/β-catenin signaling pathway among the four GSC cultures. The Western blot analysis of cytoplasmic lysates showed a marked increase in AXIN1 and TNKS1/2 protein levels in all four cultures (Figure 2a), indicating that G007-LK acts through TNKS1/2 to stabilize AXIN levels, which has been reported elsewhere [17]. To study the effect of G007-LK on the level and localization of β-catenin, we performed Western blot analysis of cytoplasmic and nuclear fractions. The analysis showed no consistent change in active β-catenin levels in either the cytoplasm or the nucleus in the GSC cultures (Figure 2a,b). We then investigated the regulation of the well-established WNT/β-catenin target genes AXIN2, DKK1, and NKD2 upon G007-LK treatment. This revealed that three out of four GSC cultures (T0965, T1008 and T2609) showed decreased expression of one or more of the three WNT/β-catenin targets (Figure 2c). In summary, we found that G007-LK stabilized AXIN1 and reduced the expression of WNT target genes in three out of the four cultures, but it did not affect the protein expression of β-catenin.

### 2.3. G007-LK Stabilizes AMOT/AMOTL2 and Reduces the Expression of YAP/TAZ Target Genes

**Figure 3:**
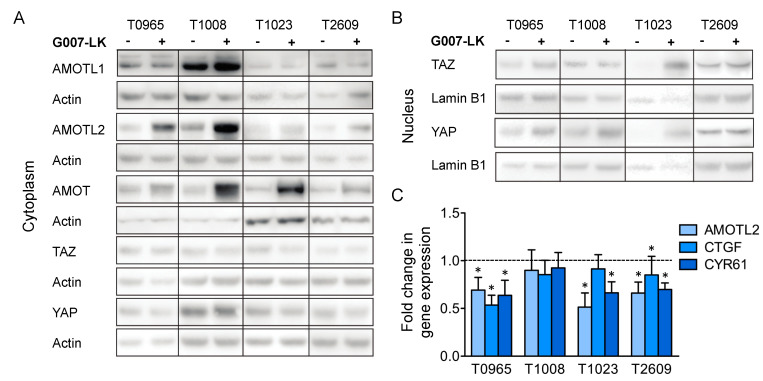
G007-LK stabilizes AMOT/AMOTL2 and reduces the expression of YAP/TAZ target genes. The effect of G007-LK treatment on the (**A**) cytoplasmic and (**B**) nuclear levels of YAP/TAZ signaling proteins; (**C**) Fold changes in gene expression of YAP/TAZ target genes, as assessed by qPCR upon treatment with G007-LK. For both analyses, GSC cultures were treated for 72 h with G007-LK (500 nM) or DMSO (0.01%). Values are relative to those of the DMSO control and are expressed as the fold changes from the DMSO control. The results are presented as the mean ± SD. * *p* < 0.05.

As G007-LK has been shown to regulate Hippo signaling [26,29], we further examined the effect of G007-LK on the expression of central proteins in the Hippo signaling pathway. The Western blot analysis of cytoplasmic lysates showed the stabilization of the YAP/TAZ regulators AMOT and AMOTL2 but not AMOTL1 (Figure 3a). We did not detect any changes in the cytoplasmic levels of TAZ and YAP. However, in three of the cell cultures, T0965, T1008 and T1023, there was a moderate increase in nuclear YAP levels upon G007-LK exposure (Figure 3b). Interestingly, T1023, the culture that did not show a change in the regulation of WNT/β-catenin target genes in our assay, was the only culture that showed an increase in both nuclear TAZ and YAP upon G007-LK treatment (Figure 3b). Next, we investigated the regulation of the YAP/TAZ target genes AMOTL2, CTGF and CYR61 by quantitative real-time PCR (qPCR). The results revealed the significant downregulation of two or three target genes in three of the GSC cultures (T0965, T1023 and T2609). In summary, consistently with previous data [26,29], we found that G007-LK stabilized the AMOT and AMOTL2 proteins and reduced the expression of YAP/TAZ target genes in three out of the four cultures, indicating a decrease in YAP/TAZ signaling. However, instead of decreasing the level of nuclear transcription factors, G007-LK treatment moderately increased the levels of nuclear YAP in three out of four cultures and increased the nuclear levels of both TAZ and YAP in T1023 cells.

### 2.4. Global Gene Expression Analysis Reveals That GSC Cultures Cluster Based on Their G007-LK Sensitivity

To explore the molecular identity of the GSC cultures and changes induced by G007-LK, we performed a global gene expression analysis of cultures following G007-LK (500 nM) or DMSO (0.01%) treatment for ten days. Interestingly, unsupervised hierarchical clustering analysis identified an expression pattern in line with the G007-LK sensitivity; the most sensitive cultures (T0965 and T1008) clustered apart from the least sensitive cultures (T1023 and T2609; Figure S1a). The least sensitive cultures displayed significantly higher expression of genes related to GBM progression and GSC proliferation and stemness, including SPARCL1, HOPX, ID1, ID3, CPE and CXRC4 (Table S1) [36,37,38,39]. However, when analyzing the cultures based on GSC subtypes [40,41], they all displayed a proneural rather than a mesenchymal signature (Figure S1b). The typical GSC stemness markers, such as SOX2, SOX9 and NES, were highly expressed in all four cultures (Figure S1c).

### 2.5. G007-LK Alters the Expression of HES Transcription Factors and Induces Apoptosis in GSC Cultures without Affecting the Cell Cycle

**Figure 4:**
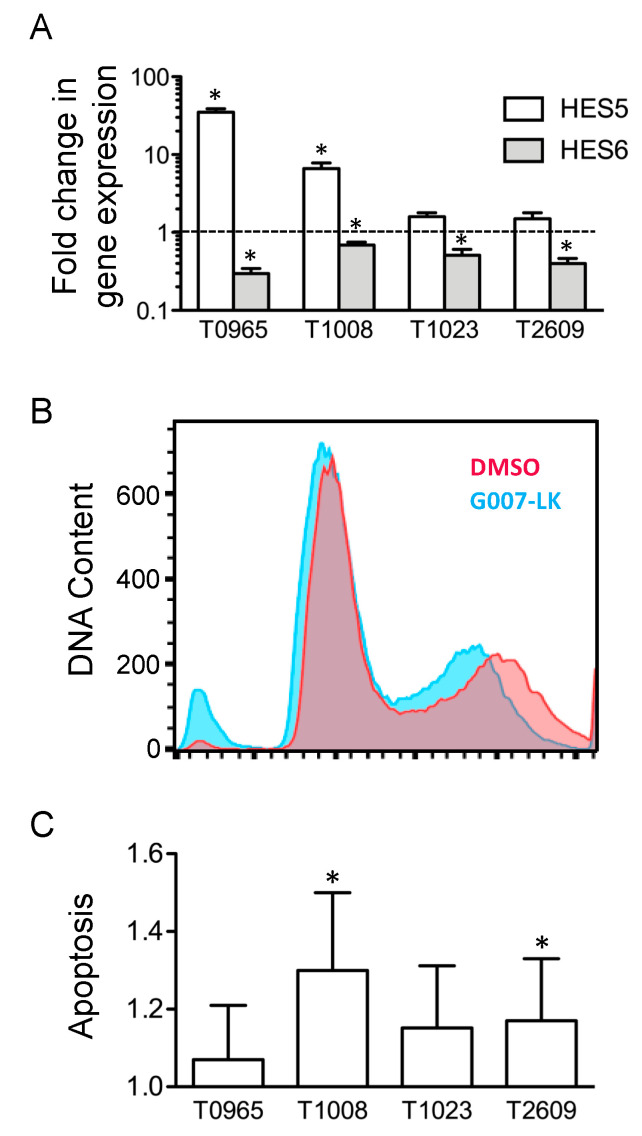
G007-LK alters the expression of HES transcription factors and induces apoptosis in primary GSC cultures without affecting the cell cycle. (**A**) Fold change in gene expression, as assessed by qPCR upon treatment with G007-LK (500 nM) for 72 h. Values are relative to those of the DMSO control (0.01%) and illustrated on a log10 scale. (**B**) Cell cycle histogram showing DNA content after ten days of treatment. Blue: G007-LK (500 nM); Red: DMSO control (0.01%). The Y-axis shows relative fluorescence. Data from T0965 are shown as a representative example. (**C**) Apoptosis in four GSC cultures after 48 h of treatment with G007-LK (500 nM). Values are expressed as the fold changes from the DMSO control (0.01%). The results are presented as the mean ± SD. * *p* < 0.05.

Finally, we extracted genes differentially expressed following G007-LK treatment. This revealed the differential expression of genes involved in proliferation, the cell cycle, and ubiquitination, as well as altered expression of the transcription factors hairy and enhancer of split (HES) 5 and 6 (Table S2). Confirmation by qPCR showed that HES6 was downregulated 2.1 ± 0.8-fold upon treatment with G007-LK; HES5 was upregulated by an average of 9.4-fold, but there was a large variation between the cultures, spanning from 1.5-fold upregulation in T2609 to 35.1-fold upregulation in T0965 (Figure 4a).

As the microarray identified the differential expression of genes related to the cell cycle, we sought to explore the effect of G007-LK on GSC cell cycling. Although there was a shift towards a narrower G2/S peak, the cell cycle analysis showed no change in the fraction of cells entering the cell cycle. However, the analysis did reveal an increase in DNA fragments, which was consistent with an increase in apoptosis (Figure 4b). We therefore examined whether G007-LK induced apoptosis in GSCs. Thus, we treated the same four GSC cultures with G007-LK (500 nM) or DMSO (0.01%) for 48 h and then assessed the amount of DNA fragmentation as an indicator of apoptosis. Although all four GSC cultures showed an increase in apoptosis, the increase was statistically significant (*p* < 0.05) in only two cultures (Figure 4c).

### 2.6. Cotreatment with Temozolomide Reduces GSCs’ Sphere-Forming Capacity

**Figure 5:**
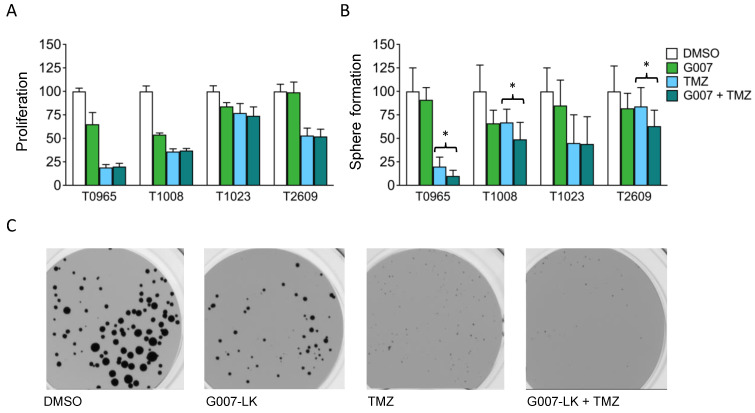
Cotreatment with temozolomide reduces the sphere-forming capacity of primary GSC cultures. (**A**) Proliferation of GSCs treated with temozolomide (TMZ) or with G007-LK/TMZ cotreatment; (**B**) Sphere formation by GSCs treated with TMZ or with G007-LK/TMZ cotreatment; (**C**) Images of T0965 spheres after 14 days of treatment. TMZ: 100 μM; G007: 500 nM; G007-LK/TMZ cotreatment: 500 nM G007-LK + 100 μM TMZ. Proliferation was measured by XTT assays and sphere formation as the total number of spheres formed in sphere formation assays, both after 14 days of treatment. Values are expressed as percentages relative to the DMSO (0.2%) control. The results are presented as the mean ± SD. * *p* < 0.05.

TMZ is an alkylating chemotherapy drug used as a first-line treatment for GBM. Synergy with conventional treatment is an attractive low threshold entry point for new therapeutic agents. We cotreated GSCs with G007-LK and TMZ for 14 days and assessed their combined effect on proliferation and sphere formation. The four GSC cultures displayed individual response patterns to TMZ treatment, with a significant reduction in proliferation found at 6.4–100 µM (Figure S2a). This response pattern was highly correlated with the promoter methylation status of MGMT (r^2^ = 0.936; Figure S2b,c). For the inhibition of proliferation, cotreatment with G007-LK was not more effective than TMZ alone (Figure 5a). However, when assessing sphere formation, cotreatment caused an additive reduction in the number of spheres formed in three out of the four GSC cultures compared to that observed following treatment with TMZ alone (Figure 5b,c).

## 3. Discussion

To our knowledge, this is the first investigation of the effect of a TNKS-specific inhibitor on primary GSC cultures. We found that in vitro treatment with G007-LK reduced the proliferation of and sphere formation by GSCs, and our results indicate that G007-LK can potentiate the activity of the chemotherapeutic drug TMZ, which is currently the standard chemotherapy for GBM. G007-LK demonstrated specific TNKS inhibition through the stabilization of TNKS1/2, AXIN1 and AMOT/AMOTL2 in all four cell cultures. Although we did not detect decreased levels of nuclear β-catenin or YAP/TAZ upon treatment with G007-LK, G007-LK did reduce the expression of typical WNT/β-catenin and YAP/TAZ target genes.

The study is based on in vitro experiments performed with primary GSC cultures. The tumor sphere culturing conditions have been shown to preserve tumorigenicity, genotype, and other patient-specific characteristics of individual GBMs [7,8,42]. We have previously demonstrated that GSC cultures between passages two and ten maintain high expression of CSC markers as well as the overall phenotype and tumorigenicity of GSCs [8]. Thus, we have worked with cell cultures at the lowest possible passages. High-passage cell lines cultured with serum, such as COLO 320DM, exhibit genotypes and phenotypes that are radically different from those of the original tumor. Moreover, COLO 320DM cells are hemizygous for a truncating APC mutation, leading to constitutive WNT/β-catenin signaling. However, we found that G007-LK reduced proliferation at comparable levels in COLO 320DM and in our low-passage primary GSCs.

In the GSC cultures, we did not find a consistent alteration of the level of active β-catenin, either in the nucleus or in the cytoplasm. However, G007-LK reduced the expression of key WNT/β-catenin target genes in three out of four tumors. In accordance with these results, we have previously found that G007-LK can regulate WNT/β-catenin target genes without a detectable change in β-catenin [43]. We also observed that while G007-LK stabilized AMOT proteins and reduced the expression of YAP/TAZ target genes, it caused a moderate accumulation of YAP protein in the nucleus. Interestingly, in a recent publication, we showed that G007-LK treatment induces the same pattern of nuclear YAP accumulation and YAP target gene reduction in both melanoma (B16-F10) and embryonic kidney (HEK293) cell lines [27]. Through confocal imaging, we demonstrated that G007-LK treatment induced the aggregation of puncta with colocalized AMOTL1-YAP and AMOTL2-YAP. This suggests that YAP’s regulation of target gene expression is more complex than what has previously been described [29,44] and may involve the inactivation of YAP-mediated transcriptional control.

There is evidence of crosstalk and reciprocal regulation between the WNT/β-catenin and Hippo signaling pathways [45,46,47]. In addition, YAP/TAZ and β-catenin/TCF/LEF have been shown to work together in regulating target gene induction in the developing heart [48]. Although this has not been demonstrated in brain cancer, YAP and TAZ are frequently upregulated in glioma [49,50], and studies have shown that TAZ is required for the FZD7-induced proliferation of glioma cells [51], while TAZ inhibition leads to senescence and growth arrest [52].

The WNT/β-catenin signaling pathway exhibits extensive crosstalk with a number of pathways in addition to the Hippo pathway [15]. We found that G007-LK altered the expression of the transcription factors HES5 and HES6, which commonly act as NOTCH effectors. However, HES5 is also a WNT/β-catenin signaling target gene [53] and tumor suppressor [54,55], while HES6 has been shown to be an oncogene [56,57] and activator of WNT/β-catenin signaling [58]. Thus, the upregulation of HES5 and downregulation of HES6 are consistent with a reduction in WNT/β-catenin signaling, as well as a diminished oncogenic profile. Interestingly, a recent study identified NOTCH receptors as targets of tankyrase-mediated degradation, indicating that the effect could be independent of WNT [59].

An important finding of this study was that in three out of four GSC cultures, G007-LK cotreatment with TMZ reduced sphere formation but not proliferation, compared to TMZ treatment alone. This is in line with the findings of Yi et al., suggesting that WNT/β-catenin signaling may contribute to TMZ resistance [60]. Interestingly, a study from 2015 found no additive effect on cell toxicity when combining G007-LK and TMZ, but it did not test the effect of treatment on sphere formation. In addition, the commercial cell lines employed, of which one was derived from GBM, showed little to no cell toxicity response upon treatment with G007-LK, even at high concentrations (up to 20 times higher than the highest dose used in this study) [61]. This is distinct from our data, which were derived using primary GSC cultures; we observed a reduction in proliferation and sphere formation in most cultures at a dose of 100 nM G007-LK, which is close to the half maximal inhibitory concentration (IC50) value reported for this compound [62]. One explanation for this disparity could be that exposure to serum changes the WNT profile of primary tumor cells, as reported by Zhang and colleagues [9].

Global gene expression analysis revealed that the most G007-LK-sensitive cultures (T0965 and T1008) clustered separately from the least G007-LK-sensitive cultures (T1023 and T2609). The least sensitive cultures expressed genes playing a role in GSC maintenance and related to the survival of GBM. Interestingly, T1023 and T2609 were also less sensitive to TMZ than T0965 and T1008 (Figure S2a). These differences in drug sensitivity between patient-derived GSC cultures may be related to a more generalized drug sensitivity profile [63].

A weakness of this study is that it is entirely based on in vitro data, so it is not directly translatable to the in vivo setting. We are currently developing and testing G007-LK analogs with enhanced bioavailability that are showing promising properties compatible with blood–brain barrier penetration [64]. Upon the successful validation of transport across the blood–brain barrier in vivo, we will initiate follow-up experiments against GBM in mouse models.

With the growing recognition of the importance of signaling pathways in cancer, a number of clinical trials are underway to assess the safety and clinical efficacy of targeting the WNT/β-catenin pathway in human patients [65,66]. To date, no clinical trial has included brain cancer patients. The findings in this study indicate that tankyrase inhibition could be an effective supplement to the current treatment regimen in these patients, possibly through affecting WNT/β-catenin and Hippo signaling. Therefore, future studies should investigate the efficacy of in vivo TNKS inhibition on GBM malignancy.

## 4. Materials and Methods

### 4.1. Biopsies and Cell Culturing

Biopsy specimens were obtained from four informed and consenting patients undergoing surgery for GBM and two patients who underwent surgery for medically intractable temporal lobe epilepsy; the tissues were used to establish four primary GSC cultures (T0965, T1008, T1023 and T2609) and two primary ahNSC cultures (H1515 and H1517). We have previously characterized all four GSC cultures and shown that they contain a high fraction of cells expressing stem cell markers (SOX2 and CD133), have the ability to differentiate upon the removal of growth factors, and form tumors upon orthotopic xenografting [8,33,34,35]. Patient-derived ahNSC cultures have previously been thoroughly characterized [6]. All subjects gave their informed consent for inclusion before they participated in the study. The study was conducted in accordance with the Declaration of Helsinki, and the protocol was approved by the Norwegian Regional Committee for Medical Research Ethics (identification code 07321b, May 2008). Histopathological diagnosis and grading were performed by a neuropathologist according to the WHO classification scheme. All the samples were IDH1/2 wild type (Table S3).

Biopsies were kept in ice-cold Leibowitz-15 medium (L-15; Invitrogen, Carlsbad, CA, USA) until the cells were isolated. The cells were isolated mechanically and enzymatically with trypsin-EDTA (Invitrogen), blocked with 2 mg/mL of human albumin (Octapharma Pharmazeutika Produktionsges, Vienna, Austria) and washed twice in L-15. The isolated cells were cultured in serum-free Dulbecco’s modified Eagle’s medium (DMEM; Gibco, Gran Island, NY, USA) containing 10 ng/mL of bFGF, 20 ng/mL of EGF (both from R&D Systems, Minneapolis, MN, USA), 1% 10,000 U/mL penicillin/10,000 µg/mL streptomycin (Lonza, Basel, Switzerland), 1 ng/mL of heparin (Leo Pharma, Copenhagen, Denmark), 8 mM HEPES (Lonza), and 1:50 B27-supplement (Gibco), as previously described [8]. The cells were plated in nontreated cell culture flasks (Nunc, Thermo Fisher Scientific, Waltham, MA, USA). This culturing method is referred to as sphere-forming culture conditions. For ahNSCs, trypsin-EDTA was replaced with papain (Worthington Biochemical Co, Lakewood, NJ, USA).

The cell line COLO 320DM (ATCC^®^ CCL-220TM) was cultured under two different conditions. When grown as spheres, COLO 320DM was cultured identically to the GSC cultures (see above). When grown under adherent culture conditions, a medium containing RPMI-1640 (Life Technologies; Carlsbad, CA, USA), 10% fetal bovine serum (FBS; PAA laboratories GmbH, Pasching, Austria), and 1% 10,000 U/mL penicillin/10,000 µg/mL streptomycin (Lonza) was used, and the cells were plated in treated cell culture flasks (Nunc). Passaging was performed as described above for the GSC cultures. Cell culture assays using primary cells were performed between passages two and 15. G007-LK was custom synthesized at ChemRoyal Inc., Tucker, GA, USA. The IC50 of G007-LK is 46 nM for TNKS1 and 25 nM for TNKS2 [62].

### 4.2. Proliferation Assay

Cells were plated at a density of 500 cells per well in a 96-well plate for suspension cells (Sarstedt, Nümbrecht, Germany) and treated for 14 days with G007-LK and/or TMZ. Control cells were treated with dimethyl sulfoxide (DMSO; Sigma-Aldrich, St. Louis, MO, USA). Proliferation was subsequently assessed using the Cell Proliferation Kit II XTT (Roche, Basel, Switzerland) according to the manufacturer’s protocol. The cells were incubated with the XTT solution overnight before the absorbance was analyzed using a microplate reader. Differences between the cells treated with G007-LK or TMZ and DMSO were assessed with an unpaired two-tailed Student’s *t*-test (Excel 14.6.6, Microsoft Office, Redmond, WA, USA), and differences between the cells treated with TMZ and those that underwent TMZ/G007-LK cotreatment were assessed by one-way ANOVA with Tukey’s multiple comparison test (Prism 5.0a, GraphPad Software, San Diego, CA, USA).

### 4.3. Sphere Formation Assay

Cells were plated at a density of 500 cells per well in a 96-well plate for suspension cells (Sarstedt, Nümbrecht, Germany) and treated for 14 days with G007-LK and/or TMZ. Control cells were treated with DMSO. Sphere formation was subsequently measured as the number of spheres in each well using an automated colony counter (Gelcount, Oxford Optronics, Abingdon, UK). Only spheres with a diameter > 50 µM were counted. Differences between the cells treated with G007-LK and DMSO were assessed with an unpaired two-tailed Student’s t-test (Excel 14.6.6, Microsoft Office), and differences between the cells treated with TMZ and those that underwent TMZ/G007-LK cotreatment were assessed by one-way ANOVA with Tukey’s multiple comparison test (Prism 5.0a, GraphPad Software).

### 4.4. Apoptosis Assay

Cells were plated at a density of 10,000 cells per well in V-shaped 96-well plates (Sarstedt) and cultured for 48 h with G007-LK (500 nM) or DMSO (0.01%). Apoptosis was then measured using the Cell Death Detection ELISAPLUS assay (Roche) and the EnVision microplate reader (PerkinElmer, Waltham, MA, USA). Differences between the cells treated with G007-LK and DMSO were assessed with an unpaired two-tailed Student’s t-test (Excel 14.6.6, Microsoft Office).

### 4.5. RNA Extraction and Quantitative Real-Time PCR

Cells were cultured for ten days with G007-LK (500 nM) or DMSO (0.01%) at a density of 500,000 cells/mL in nontreated cell culture flasks (Nunc). Total RNA was extracted using Qiazol and the RNeasy Micro Kit (Qiagen GmbH, Hilden, Germany). RNA concentrations were determined using the Nanodrop spectrophotometer (Thermo Fisher Scientific), and they were analyzed for quality using the Experion System (Bio-Rad Laboratories, Hercules, CA, USA). Only samples with an RNA quality indicator score > 8.0 were included. For quantitative real-time PCR (qPCR) analysis, the High-Capacity cDNA Reverse Transcription Kit, TaqMan Universal PCR Master Mix, TaqMan oligonucleotide primers and probes, and the ABI Prism Detection System and software (Hs00170014_m1(CTGF), Hs00155479_m1(CYR61), Hs01048101_m1(AMOTL2), Hs01387463_g1 (HES5), Hs00936587_g1(HES6), Hs00610344(AXIN2), Hs00183740_m1(DKK1) and Hs01548773_m1(NKD1), all from Applied Biosystems, Waltham, MA, USA) were used according to the manufacturers’ instructions. Human β-Actin (Hs99999903_m1, TaqMan endogenous control reagents, Applied Biosystems) was used as a housekeeping gene. The thermal cycling conditions were 2 min at 50 °C and 10 min at 94.5 °C, followed by 40 cycles of 30 s at 97 °C and 1 min at 59.7 °C. The relative gene expression levels were calculated using the standard curve method [67].

### 4.6. Microarray Analysis

Cells were cultured for ten days with G007-LK (500 nM) or DMSO (0.01%) at a density of 500,000 cells/mL in nontreated cell culture flasks (Nunc). Single technical replicates of RNA samples from each tumor culture (four biological replicates) were run on a HumanHT-12 chip (Illumina, San Diego, CA, USA). Analysis and statistics were performed using J-Express (Molmine, Bergen, Norway). The unsupervised hierarchical clustering was performed using complete linkage and Pearson correlation. The molecular subtype was defined according to Phillips and Mao [40,41]. Differential gene expression analysis comparing the most (T0956 and T1008) and least G007-LK-sensitive cultures (T1023 and T2609) was carried out using Rank product analysis (cut-off *q* < 5%). The gene expression changes induced by G007-LK were identified by performing paired significance analysis of microarrays (SAM; cut-off *q* < 10%).

### 4.7. Western Blotting

Cells were cultured for 72 h with G007-LK (500 nM) or DMSO (0.01%) at a density of 500,000 cells/mL in nontreated cell culture flasks (Nunc). Cell extracts were made by adding cold RIPA buffer (Thermo Fisher Scientific) containing protease inhibitors (Protease Inhibitor Cocktail Tablets, Roche) and phosphatase inhibitors (PhosStop Tablets, Sigma-Aldrich) to frozen cell pellets, following the manufacturer’s protocol for the preparation of cell extracts from adherent cells. For cytoplasmic and nuclear extracts, proteins were isolated from frozen cell pellets using the Thermo NE-PER Nuclear and Cytoplasmic Extraction kit (Thermo Fisher Scientific) per the manufacturer’s instructions. The protein concentrations were determined using the Pierce BCA Protein Assay Kit (Thermo Fisher Scientific), and 10 μg of protein was loaded onto gels (Novex Bis-Tris or Tris-Acetate gels, Life Technologies) along with a PageRuler prestained protein ladder (Fermentas, Thermo Fisher Scientific). Subsequently, the gels were analyzed using Novex electrophoresis chambers (Life Technologies). The proteins were transferred to 0.2 μm nitrocellulose membranes (Novex, Life Technologies), and then they were blocked with 5% milk (AppliChem BmbH, Darmstadt, Germany) and 0.05% Tween-20 in TBS (Medicago, Uppsala, Sweden) for 1 h. The membranes were then stained with primary (4 °C overnight with rocking in 1% milk and 0.05% Tween-20 in TBS) and secondary antibodies (1 h at room temperature with rocking in 5% milk and 0.05% Tween-20 in TBS). Bands were visualized using ECL Prime Western Blotting Detection Reagent (GE Healthcare, Pittsburgh, PA, USA) in a ChemiDoc Touch Imaging System (Bio-Rad Laboratories) developer. The Image Lab Software (Bio-Rad Laboratories) was used to quantify bands (normalized against loading controls). The quantified protein immunoblot ratios (protein vs. loading control) for the Western blot analyses presented in Figure 2 and Figure 3 are available in Figure S3 (Figure 2) and Figure S4 (Figure 3). Detailed information about the Western blotting can be found in Figures S5 and S6.

The primary antibodies used were monoclonal rabbit anti-nonphospho (active) β-catenin (1:1000; D13A1, Cell Signaling, Danvers, MA, USA), monoclonal mouse anti-β-catenin (1:1000; 610154, BD Biosciences, San Jose, CA, USA), monoclonal anti-AXIN1 (1:1000; C76H11, Cell Signaling), polyclonal anti-TNKS1/2 (1:250; H-350, Santa Cruz Biotechnology, Dallas, TX, USA), YAP (sc-101199, Santa Cruz Biotechnology), TAZ (HPA007415, Sigma Aldrich), AMOT (sc-166924, Santa Cruz Biotechnology), AMOTL1 (PA5-42267, Thermo Fisher Scientific) and AMOTL2 (PA5-78770, Thermo Fisher Scientific). Monoclonal rabbit anti-actin (1:2000; A5441, Sigma-Aldrich) and polyclonal rabbit anti-lamin B1 (1:1000; ab16048, Abcam, Cambridge, UK) were used as loading controls.

### 4.8. Cell Cycle Assay

Cells were cultured for ten days with G007-LK (500 nM) or DMSO (0.01%) at a density of 500,000 cells/mL in nontreated cell culture flasks (Nunc). During the last 24 h, the samples were incubated with 5-ethynyl-2’-deoxyuridine (EdU; 10 μM) from the Click-iT EdU Alexa Fluor 488 Cell Proliferation Assay Kit (Invitrogen). Analysis was performed using an LSRII flow cytometer (BD Biosciences) according to the manufacturer’s recommendations, and the experiments were twice repeated independently.

### 4.9. MGMT Promoter Methylation Status

Genomic DNA was isolated from cells using the Maxwell 16 Cell DNA Purification Kit and the Maxwell 16 Instrument (Promega, Madison, WI, United States) before treatment with the EpiTect Bisulfite Kit (Qiagen GmbH). This treatment of the DNA results in the conversion of unmethylated cytosine residues into uracil, leaving the methylated cytosines unchanged. Subsequently, qPCR was performed using the MGMT Pyro Kit (Qiagen GmbH) according to the manufacturer’s instructions. The samples were then processed in the PyroMark Q24 system (Qiagen GmbH), and the obtained data were analyzed with the PyroMark CpG Software (Qiagen GmbH). MGMT promoter methylation was calculated as an average for the four CpG islands. Cultures with an average of ≥10% were considered to be MGMT methylated.

### 4.10. Statistics

Data are presented as means ± standard deviations. The differences between cells treated with G007-LK or TMZ and DMSO were assessed with an unpaired two-tailed Student’s t-test (Excel 14.6.6, Microsoft Office), and the differences between cells treated with TMZ and those that underwent TMZ/G007-LK cotreatment were assessed by one-way ANOVA with Tukey’s multiple comparison test (Prism 5.0a, GraphPad Software). The results are based on three independent experiments unless otherwise stated. Statistical significance was determined at *p*-value < 0.05 (*).

## 5. Conclusions

This study is the first investigation of the effect of a TNKS-specific inhibitor on primary GSC cultures. We found that G007-LK reduced GSC proliferation and sphere formation in vitro and that cotreatment with TMZ led to an additive reduction in sphere formation but not proliferation, indicating that WNT/β-catenin signaling may contribute to TMZ resistance. G007-LK demonstrated specific TNKS inhibition through the stabilization of TNKS1/2, AXIN1 and AMOT/AMOTL2 in all four cell cultures, and it reduced the expression of typical WNT/β-catenin and YAP/TAZ target genes. These observations indicate that TNKS inhibition affects WNT/β-catenin and Hippo signaling and may serve as a supplement to current GBM therapy, although more work remains to determine the exact downstream mechanisms involved.

## Supplementary Materials

The following are available online at https://www.mdpi.com/2072-6694/12/6/1630/s1. Figure S1: Global gene expression analysis with hierarchical clustering, Figure S2: TMZ sensitivity and correlation with MGMT promoter methylation, Figure S3: Quantified protein immunoblot ratios (protein vs. loading control) of cytoplasmic and nuclear levels of the WNT/β-catenin signaling proteins shown in Figure 2, Figure S4: Quantified protein immunoblot ratios (protein vs. loading control) for cytoplasmic and nuclear levels for the YAP/TAZ signaling proteins shown in Figure 3, Figure S5: Detailed information about the Western blot in Figure 2a, Figure S6: Detailed information about the Western blot in Figure 3a, Table S1: Unsupervised gene expression analysis of cell cultures treated with G007 (500 nM) or DMSO (0.01%), Table S2: Results from global gene expression analysis (SAM, *q* < 10%) of GSC cultures treated with G007 (500 nM) or DMSO (0.01%), Table S3: IDH methylation status for the four patient-derived glioblastomas.
