# Supplementary material for: A Small-Molecule Tankyrase Inhibitor Reduces Glioma Stem Cell Proliferation and Sphere Formation
Source: Cancers (Basel). 2020 Jun 19;12(6):1630. doi: 10.3390/cancers12061630 (PMC7352564; doi:10.3390/cancers12061630)
Supplement: Supplementary file 1 [file cancers-12-01630-s001.pdf]

Supplementary Materials

A Small-Molecule Tankyrase Inhibitor Reduces Glioma Stem Cell Proliferation and Sphere Formation

Kirsten S. Kierulf-Vieira, Cecilie Jonsgar Sandberg, Jo Waaler, Kaja Lund, Erlend Skaga, Birthe Mikkelsen Saberniak, Ioannis Panagopoulos, Petter Brandal, Stefan Krauss, Iver Arne Langmoen and Einar Osland Vik-Mo

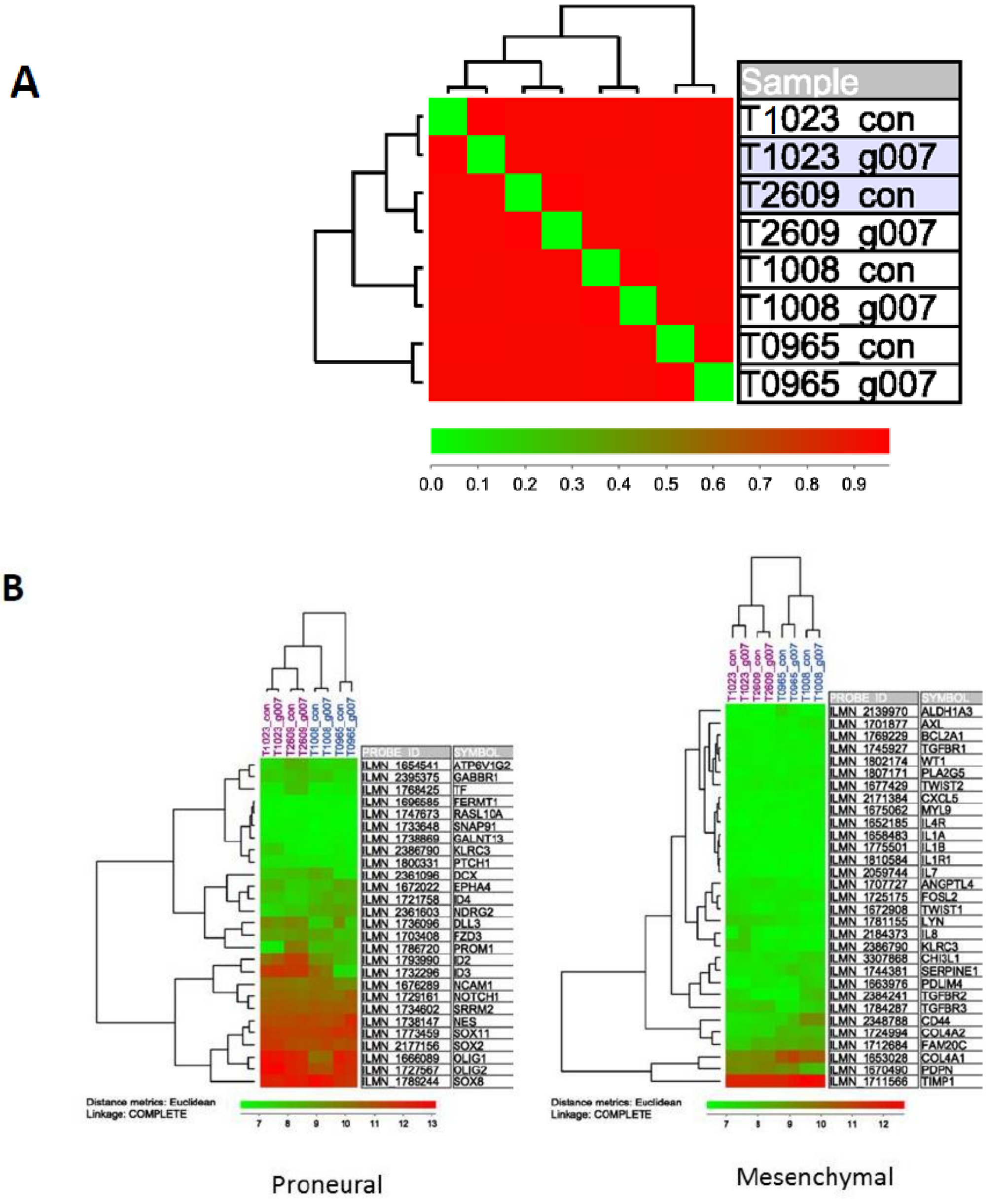

C

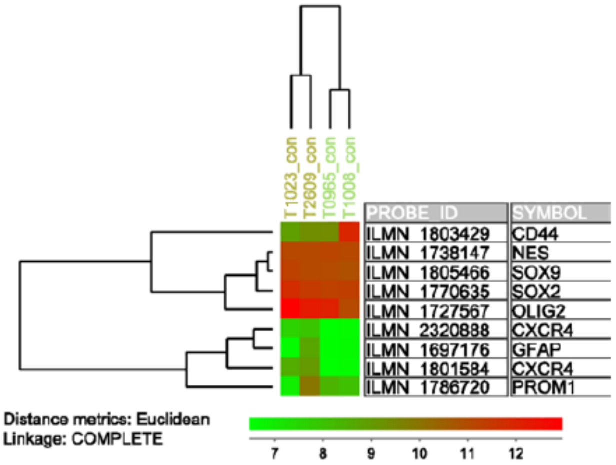

Figure S1. Global gene expression analysis with hierarchical clustering. (**A**) Unsupervised hierarchical clustering analysis of the global gene expression with distance matrix (average linkage, Pearson Correlation). (**B**) Unsupervised hierarchical clustering analysis of the gene expression representing the proneural and the mesenchymal subtypes of GSCs. (**C**) Unsupervised hierarchical clustering analysis of the gene expression representing typical GSC molecular markers.

A

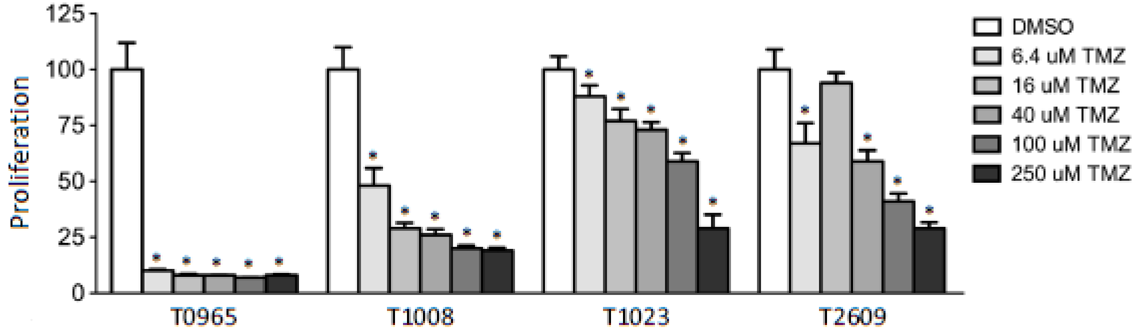

B

MGMT promoter methylation, %						
GSC Culture	CpG1	CpG2	CpG3	CpG4	Average	Methylation (10% limit)
T0965	96.4	97.0	100.0	100.0	**98.3**	+
T1008	43.7	34.9	99.7	52.7	**57.7**	+
T1023	10.2	10.6	12.3	10.8	**10.9**	+
T2609	20.4	53.8	37.4	46.5	**39.5**	+

C

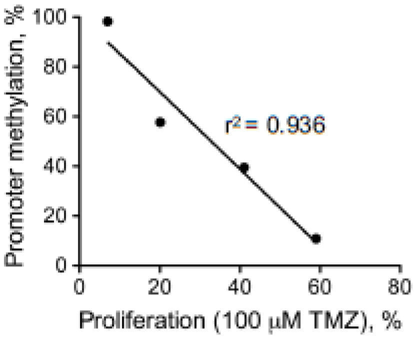

Figure S2. TMZ sensitivity and correlation with MGMT promoter methylation. (**A**) Dose-response curves for TMZ in four primary GSC cultures treated with TMZ for 14 days, presented as percentage of the DMSO control (0.5 %). Results are presented as mean ± SD. (**B**) Percentage MGMT promoter methylation calculated as an average for the four CpG islands. Cultures with an average of ≥ 10 % were considered to be MGMT methylated. (**C**) Correlation of MGMT promoter methylation to anti-proliferative effect of TMZ at 100 μM, as percentage of DMSO. * $p < 0.05$.

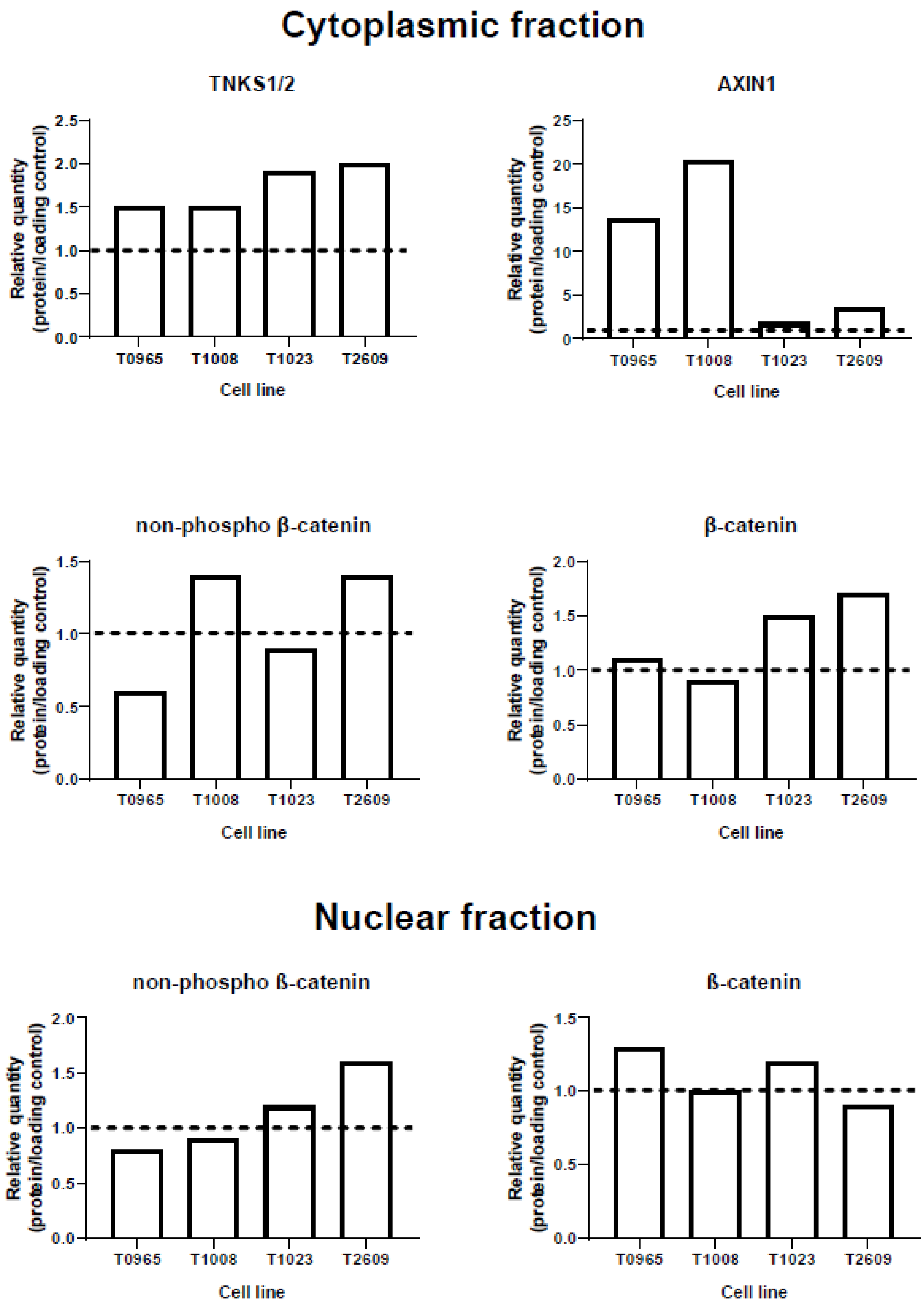

Figure S3. Quantified protein immunoblot ratios (protein vs. loading control) for cytoplasmic and nuclear levels for WNT/β-catenin signaling proteins shown in Figure 2.

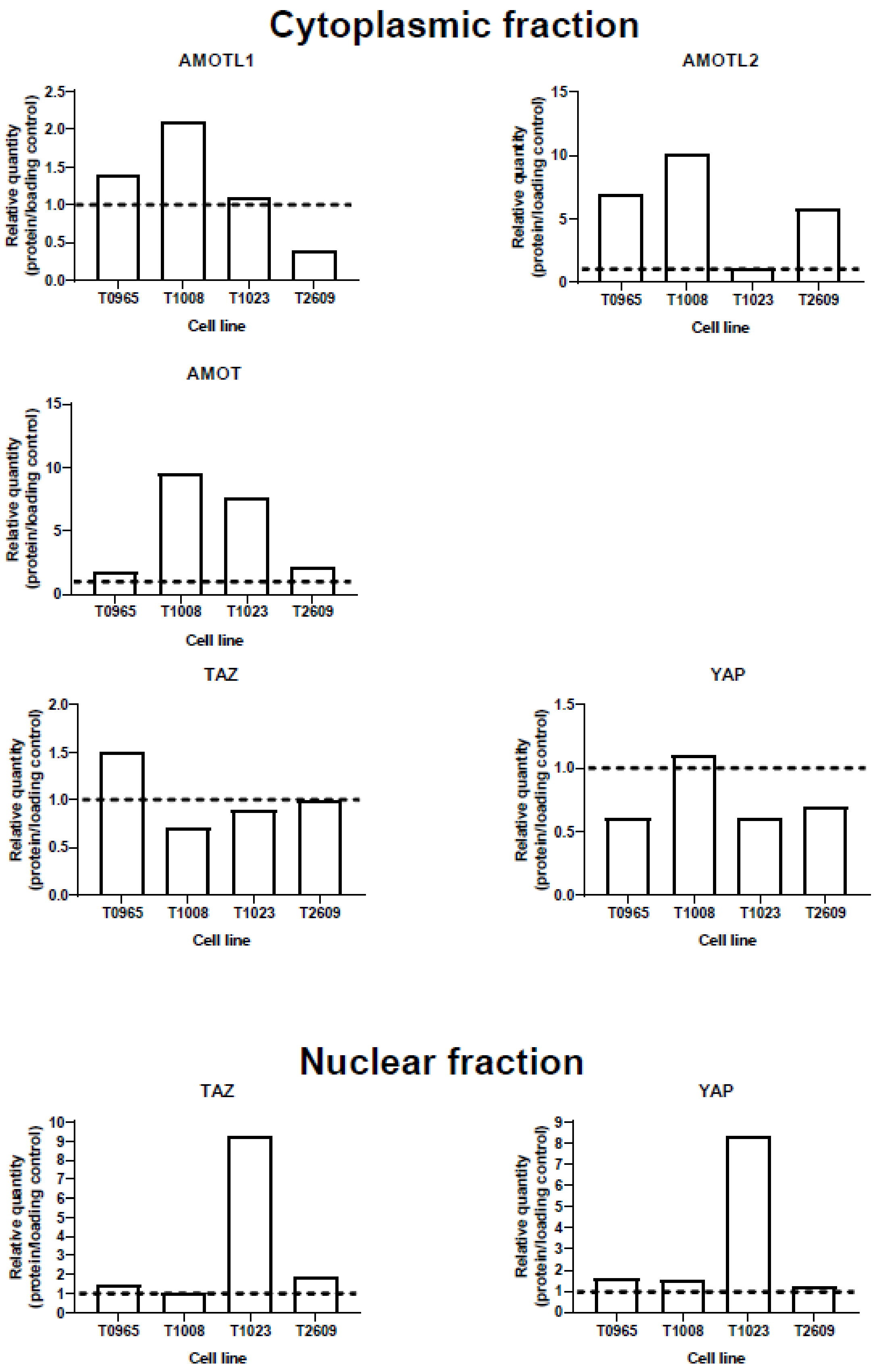

Figure S4. Quantified protein immunoblot ratios (protein vs. loading control) for cytoplasmic and nuclear levels for YAP/TAZ signaling proteins shown in Figure 3.

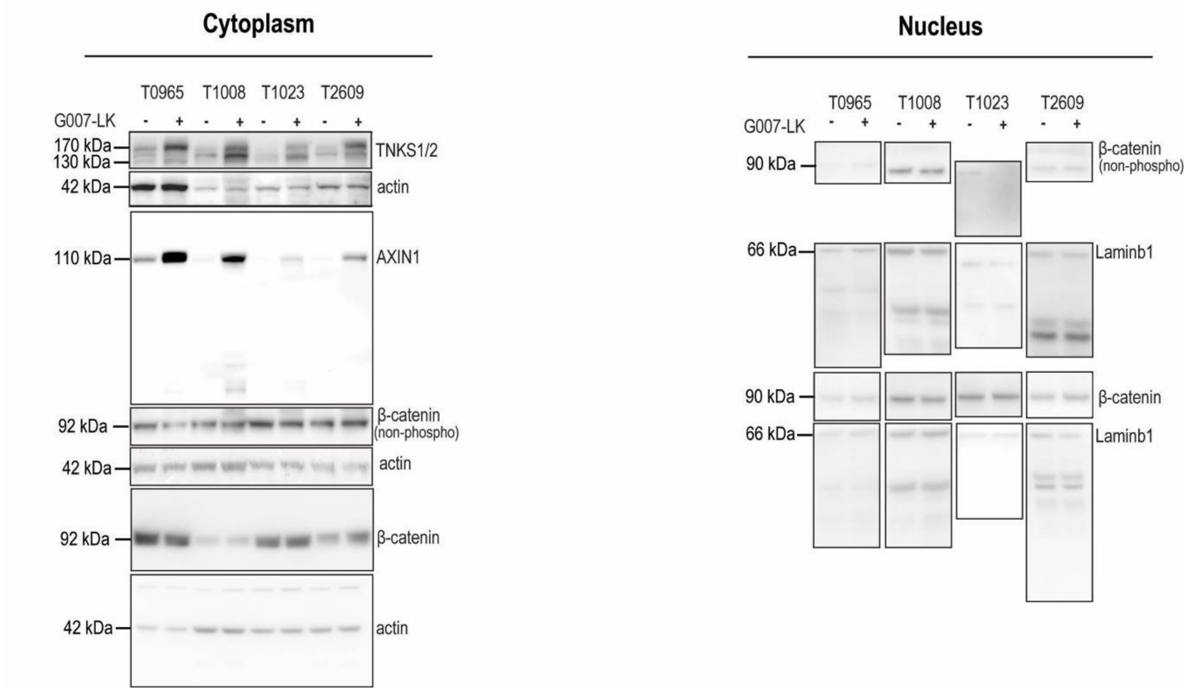

Figures S5. Detailed information about the western blot for Figure 2A.

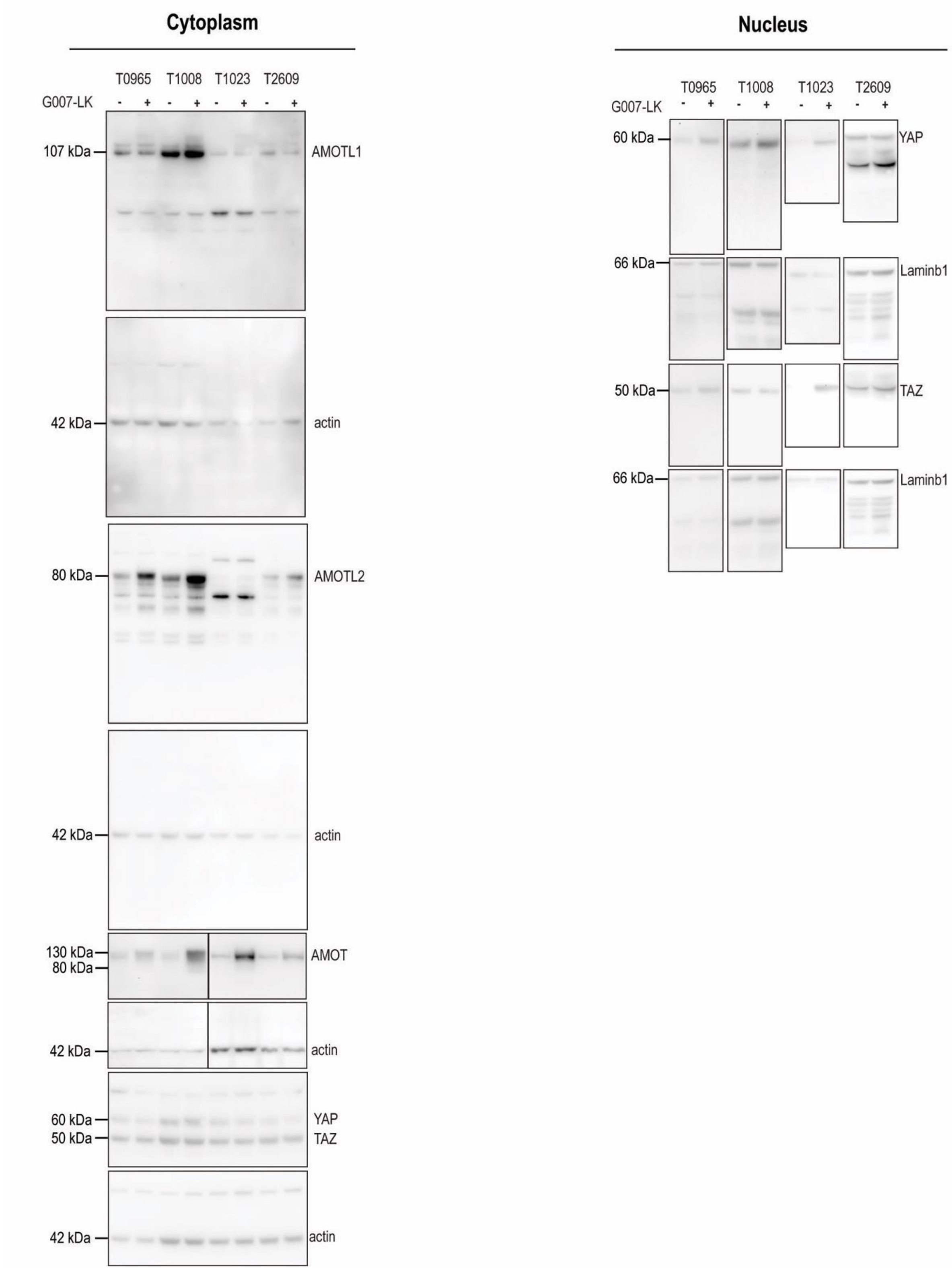

Figures S6. Detailed information about the western blot for Figure 3A.

Table S1. Unsupervised gene expression analysis of cell cultures treated with G007 (500 nM) or DMSO (0.01%).

Probe_ID	Symbol	ILMN_GENE	Entrez_Gene	Fold Change Low Sensitivity/ High Sensitivity
ILMN_1795251	SPARCL1	SPARCL1	8404	15.95
ILMN_1664861	ID1	ID1	3397	10.46
ILMN_1731374	CPE	CPE	1363	5.83
ILMN_1732296	ID3	ID3	3399	6.65
ILMN_1696657	LRRN2	LRRN2	10446	5.30
ILMN_1801584	CXCR4	CXCR4	7852	4.86
ILMN_2316236	HOPX	HOPX	84525	4.55
ILMN_1760849	NETO2	NETO2	81831	6.41
ILMN_1688722	IL13RA2	IL13RA2	3598	4.70
ILMN_1813741	KCNJ16	KCNJ16	3773	4.29
ILMN_1794742	HES5	HES5	388585	4.29
ILMN_1699980	TSPAN18	TSPAN18	90139	4.04
ILMN_2169761	CPNE8	CPNE8	144402	4.51
ILMN_1762696	FAM181A	FAM181A	90050	3.90
ILMN_1798975	EGFR	EGFR	1956	5.82
ILMN_1807969	SNCAIP	SNCAIP	9627	3.69
ILMN_1674782	LOC375295	LOC375295	375295	4.17
ILMN_1720511	LRRN1	LRRN1	57633	3.54
ILMN_1781388	PGM5	PGM5	5239	3.72
ILMN_1703178	SCG2	SCG2	7857	0.19
ILMN_1677723	ANGPT1	ANGPT1	284	0.22
ILMN_1696537	DDIT4L	DDIT4L	115265	0.21
ILMN_2345142	SULF2	SULF2	55959	0.28
ILMN_2336094	ODZ3	ODZ3	55714	0.23
ILMN_2148527	H19	H19	283120	0.23
ILMN_1674785	COL2A1	COL2A1	1280	0.25
ILMN_1656111	MYLIP	MYLIP	29116	0.30
ILMN_1733515	LOXL3	LOXL3	84695	0.26
ILMN_1728478	CXCL16	CXCL16	58191	0.28
ILMN_1797776	PRSS23	PRSS23	11098	0.27
ILMN_1769282	FRMD6	FRMD6	122786	0.29
ILMN_1765641	SEMA3A	SEMA3A	10371	0.25
ILMN_1805665	FLRT3	FLRT3	23767	0.27
ILMN_1745994	GAS7	GAS7	8522	0.18
ILMN_1685608	NPTX2	NPTX2	4885	0.24
ILMN_1801616	EMP1	EMP1	2012	0.26
ILMN_2374449	SPP1	SPP1	6696	0.24
ILMN_1784320	ELMO1	ELMO1	9844	0.22
ILMN_1751276	BDNF	BDNF	627	0.24
ILMN_1757497	VGF	VGF	7425	0.23
ILMN_1766054	ABCA1	ABCA1	19	0.21
ILMN_2188521	PVRL3	PVRL3	25945	0.18
ILMN_1780170	APOD	APOD	347	0.15

Table S2. Results from global gene expression analysis (SAM, $q < 10\%$) of GSC cultures treated with G007 (500 nM) or DMSO (0.01%).

PROBE_ID	SYMBOL	ILMN_GENE	ENTREZ_GENE_ID	q-val[i]	T0836 DMSO	T0836 G007	T0965 DMSO	T0965 G007	T1008 DMSO	T1008 G007	T1023 DMSO	T1023 G007	T2609 DMSO	T2609 G007
ILMN_1694268	HES6	HES6	55502	0	12.45	11.88	11.72	10.85	11.73	11.17	12.11	11.52	12.07	11.63
ILMN_1752394	CCNB1IP1	CCNB1IP1	57820	0	7.68	7.06	9.22	8.80	8.83	8.54	8.48	8.07	9.17	8.68
ILMN_1766446	C6orf48	C6ORF48	50854	0	7.80	7.28	8.07	7.56	7.90	7.61	7.94	7.47	7.76	7.43
ILMN_2387990	UBE2J2	UBE2J2	118424	0	7.59	7.30	8.11	7.72	7.96	7.61	8.07	7.77	8.04	7.80
ILMN_2347349	CCNB1IP1	CCNB1IP1	57820	0	8.26	7.64	10.04	9.68	9.73	9.36	9.30	8.89	9.94	9.61
ILMN_3227023	SNHG7	SNHG7	84973	0	9.21	8.91	10.74	10.15	9.67	9.40	9.59	9.06	10.02	9.63
ILMN_1707631	MED10	MED10	84246	8.69	9.59	9.26	9.52	9.29	9.84	9.55	9.95	9.40	9.65	9.24
ILMN_2403237	CHN2	CHN2	1124	8.69	8.07	7.81	7.61	7.41	8.09	7.83	7.79	7.49	7.26	7.08
ILMN_1726986	AADAT	AADAT	51166	8.69	8.93	8.66	8.96	8.70	9.27	8.93	8.90	8.70	8.85	8.65
ILMN_2391765	C6orf48	C6ORF48	50854	8.69	11.82	11.69	12.25	11.67	12.10	11.60	12.06	11.38	11.79	11.04
ILMN_1664698	UNC119	UNC119	9094	8.69	8.04	7.73	7.88	7.71	8.31	7.83	8.37	7.91	8.11	7.77
ILMN_1794742	HES5	HES5	388585	7.97	9.61	10.07	6.77	8.19	6.80	7.38	8.20	8.66	9.57	10.28

Table S3. IDH methylation status for the four patient-derived glioblastomas.

GSC Culture	IDH1 Forward	IDH1 Reverse	Type Mutation	IDH2 Forward	IDH2 Reverse	Type Mutation
**T0965**	CGT		-	AGG		-
**T1008**	CGT		-	AGG		-
**T1023**	CGT		-	AGG		-
**T2609**	CGT		-	AGG		-

© 2020 by the authors. Licensee MDPI, Basel, Switzerland. This article is an open access article distributed under the terms and conditions of the Creative Commons Attribution (CC BY) license (http://creativecommons.org/licenses/by/4.0/).
